# Unraveling Heparan Sulfate Proteoglycan Binding Motif for Cancer Cell Selectivity

**DOI:** 10.3389/fonc.2019.00843

**Published:** 2019-09-18

**Authors:** Jlenia Brunetti, Giulia Riolo, Lorenzo Depau, Elisabetta Mandarini, Andrea Bernini, Evgenia Karousou, Alberto Passi, Alessandro Pini, Luisa Bracci, Chiara Falciani

**Affiliations:** ^1^Department of Medical Biotechnologies, University of Siena, Siena, Italy; ^2^Department of Biotechnology, Chemistry and Pharmacy, University of Siena, Siena, Italy; ^3^Department of Medicine and Surgery, University of Insubria, Varese, Italy

**Keywords:** heparan sulfate proteoglycans, peptide, tumor targeting, sulfatase, oligosaccharide

## Abstract

Membrane heparan sulfate proteoglycans (HSPG) regulate cell proliferation, migration, and differentiation and are therefore considered key players in cancer cell development processes. Here, we used the NT4 peptide to investigate how the sulfation pattern of HSPG on cells drives binding specificity. NT4 is a branched peptide that binds the glycosaminoglycan (GAG) chains of HSPG. It has already been shown to inhibit growth factor-induced migration and invasiveness of cancer cells, implying antagonist binding of HSPG. The binding affinity of NT4 with recombinant HSPG showed that NT4 bound glypican-3 and -4 and, with lower affinity, syndecan-4. NT4 binding to the cancer cell membrane was inversely correlated with sulfatase expression. NT4 binding was higher in cell lines with lower expression of SULF-1 and SULF-2, which confirms the determinant role of sulfate groups for recognition by NT4. Using 8-mer and 9-mer heparan sulfate (HS) oligosaccharides with analog disaccharide composition and different sulfation sites, a possible recognition motif was identified that includes repeated 6-O-sulfates alternating with N- and/or 2-O-sulfates. Molecular modeling provided a fully descriptive picture of binding architecture, showing that sulfate groups on opposite sides of the oligosaccharide can interact with positive residues on two peptide sequences of the branched structure, thus favoring multivalent binding and explaining the high affinity and selectivity of NT4 for highly sulfated GAGs. NT4 and possibly newly selected branched peptides will be essential probes for reconstructing and unraveling binding sites for cancer-involved ligands on GAGs and will pave the way for new cancer detection and treatment options.

## Introduction

Heparan sulfate proteoglycans (HSPG) are a large family of heterogeneous molecules found in the extracellular matrix (ECM) and on the membranes of vertebrate cells. They are composed of a protein linked to sulfated glycosaminoglycan (GAG) chains, which are linear polymers of repeated disaccharide units consisting of an amino sugar and uronic acid, that can be modified with sulfate groups at various positions. HSPG can be classified by their localization as extracellular, intracellular, pericellular, and cell surface associated. Cell surface HSPG include the two families of syndecans and glypicans and betaglycan, a transmembrane proteoglycan (PG) with heparan and chondroitin sulfate chains. Glycosaminoglycan moieties in membrane-associated HSPG do not differ much in saccharide composition but are very different in sulfation pattern in terms of positions and number of sulfates (1, 2). Since membrane HSPG regulate cell proliferation, adhesion, migration, and differentiation (3, 4), they are considered key players in cancer cell development (1). This is because GAG chains of HSPG interact with a large number (>435) of extracellular regulatory proteins, such as growth factors, chemokines, and morphogens (5). Indeed, drugs directed against HSPG are being evaluated in preclinical models. For example, peptides directed against syndecan-1 have shown therapeutic promise in preclinical models of breast cancer and myeloma (6–8).

NT4 peptide is a tetrabranched peptide that binds to GAG chains of HSPG. Its branched structure, obtained by synthesizing four copies of the 13-amino-acid sequence on a branching core of lysines, makes NT4 stable to proteolytic enzymes and gives it a long half-life (9, 10). NT4 binds cell lines of different human cancers, including colon adenocarcinoma, pancreas adenocarcinoma, bladder cancer, and breast cancer (11, 12). It does not bind PgsA-745 cells (Chinese hamster ovary cell mutant), which lack GAG chains, being deficient in xylosyltransferase, the enzyme responsible for anchorage of GAG chains to the protein core (13). Tumor selectivity was very evident in surgical resections of colon, pancreas, and bladder cancer, stained with NT4 conjugated with a fluorescent probe, compared to the healthy counterparts (14–16).

NT4 peptides can be conjugated with different functional units and can selectively deliver drugs for cancer therapy or transport tracers for tumor imaging (11, 12, 15–18). Using drug-conjugated NT4, we obtained a significant reduction in tumor growth or even tumor regression (11, 14, 17), compared to animals treated with the unconjugated drug under identical conditions. NT4 transports the chemotherapeutic moiety to the cancer cell membrane and, ultimately, into the cell (14–16). In animal models of cancer, the higher concentration of the cytotoxic drug at the site of the tumor, obtained by the targeting with the peptide, showed better efficacy than the free drug (11, 14, 17). We found that the high selectivity of NT4 toward cancer cells and tissues resides in its high-affinity binding to sulfated GAGs, with preferential high-affinity binding to heparin and heparan sulfate (HS) compared to chondroitin sulfate (CS) (13, 19). Importantly, NT4 inhibited oriented migration of pancreas adenocarcinoma cells (13) as well as growth factor-induced migration and invasiveness of breast cancer cells, implying antagonist binding to HSPG (13, 20).

Here, we report how the sulfation pattern of HSPG on cells can drive binding specificity. Regardless the expression of different HSPG on cancer cells, GAG linear polymers are the only exposed HSPG moiety on the outer membrane and are responsible for specificity.

The glycoside sequence and sulfation pattern of GAGs are crucial for ligand binding and are synthesized by enzymes in the Golgi apparatus and modified by extracellular enzymes that can introduce recognition patterns for growth factors (2) and other binding proteins. The specificity of GAG–ligand interactions has been reported in several studies. For example, it has been described in the case of the fibroblast growth factor (FGF)–heparin interaction, where the key residues on FGF and GAG chains were identified (21). The FGF–HS–FGFR1 ternary complex can only be formed in the presence of 6-O-sulfate groups on HS (22, 23). Interestingly, it has been observed that short analogs of heparin, i.e., heparin oligosaccharides, featuring one or two 6-O-sulfate groups on the reducing end of glucosamine, can fully activate FGF2 signaling (24). 6-O-sulfation of HS is also reported to be necessary to prompt the response of primary fibroblasts to transforming growth factor-β1 (TGFβ1), whereas 6-O-sulfates negatively regulate Wnt signaling (25, 26).

NT4 binds a specific pattern and competes with GAG binding proteins for important biological functions like angiogenesis and migration. As such, NT4 was used here to define the fine structure of binding sites on GAG chains.

## Methods

### Peptide Synthesis

Peptides were synthesized on an automated multiple synthesizer (MultiSynTech, Germany) by standard Fmoc chemistry. NT4 was synthesized on Fmoc_4_-Lys_2_-Lys-beta-Ala-Tentagel resin (Rapp Polymer) using protected L-amino acids (Iris Biotech), DIPEA (N,N-diisopropylethylamine) (Merck), and HBTU (hexafluorophosphate benzotriazole-N,N,N',N'-tetramethyl uronium) (MultiSynTech). Pyro-Glu-O-pentachlorophenylester (Bachem, Switzerland) was used for the last coupling step. NT4-biotin was synthesized on Tentagel resin with Fmoc-Lys(biotin)-OH as the first coupling step, and Fmoc-PEG_12_-OH as the second; Fmoc-Lys(Fmoc)-OH was then used to build the tetrameric core. At the end of the coupling sequence, peptides were cleaved from the resin, deprotected, and lyophilized.

High-performance liquid chromatography (HPLC) purification was performed on a C18 Jupiter column (Phenomenex). Water with 0.1% trifluoroacetic acid (TFA) (A) and methanol (B) were used as eluents. Linear gradients over 30 min were run at flow rates of 0.8 and 4 ml/min for analytical and preparatory procedures, respectively. All compounds were also characterized on a BrukerUltraflex matrix-assisted laser desorption/ionization time-of-flight/time-of-flight (MALDI TOF/TOF) mass spectrometer.

NT4 (pyELYENKPRRPYIL)_4_K_2_K-beta-Ala MS: *m*/*z* calculated for C_333_H_519_N_91_O_81_ [M+H]^+^ was 7,094.24; detected 7,095.15. HPLC RT (from 80 to 20%A) 26.63 min. NT4-biotin (pyELYENKPRRPYIL)_4_K_2_K-PEG_12_-K(biotin) MS: *m*/*z* calculated for C_373_H_594_N_96_O_95_S [M+H]^+^ was 7,976.35; detected 7,978.72. HPLC RT (from 80 to 20%A) was 26.99 min.

### Cell Lines

PANC-1 human pancreas adenocarcinoma, HT-29 human colon adenocarcinoma, and MCF-7 and MDA-MB-231 human breast adenocarcinoma cells were grown in the recommended American Type Culture Collection (ATCC) media, supplemented with 10% fetal calf serum, 200 μg/ml glutamine, 100 μg/ml streptomycin, 60 μg/ml penicillin, and maintained at 37°C, 5% CO_2_. Cell lines were purchased from ATCC, and cell profiling was analyzed to authenticate human cell lines (BMR Genomics).

### Flow Cytometry

All experiments were performed using 2 × 10^5^ cells in 96-well U-bottom plates. All dilutions were performed in phosphate-buffered saline (PBS), containing 5 mM EDTA and 1% bovine serum albumin (BSA).

### NT4 Binding

Cells were incubated with 1 μM NT4-biotin for 30 min at room temperature and then incubated with 1 μg/ml streptavidin–fluorescein isothiocyanate (FITC). For heparinase treatment, cells were incubated for 1 h at 37°C on the plates with 0.03 IU/ml heparinase I/III blend (Sigma Aldrich), and then harvested and incubated with the same concentration of heparinase in suspension for an additional hour at 37°C before NT4 staining. All experiments were repeated two times. *P* values were calculated using a two-tailed Student *t*-test and GraphPad Prism 5.0 software.

### Real-Time Polymerase Chain Reaction (qRT-PCR)

Total RNA samples were extracted from different human cancer cells (1 × 10^6^ cells) with TRIzol (Invitrogen, Milan, Italy). For quantitative RT-PCR, RNA samples were retrotranscribed using the High-Capacity cDNA Synthesis Kit (Applied Biosystems, Monza, Italy) and amplified on an Abi Prism 7000 instrument (Applied Biosystems, Monza, Italy) using the TaqMan Universal PCR Master Mix (Applied Biosystems) following the manufacturer's instructions.

The following human TaqMan gene expression assays were used: glypican-3 GPC3 (Hs00170471_ml), glypican-4 GPC4 (Hs00155059_m1), syndecan-3 SDC3 (Hs00206320_m1), syndecan-4 SDC4 (Hs00161617_m1), and β-actin (Hs99999903_m1). Fluorescent signals generated during PCR amplifications were monitored and analyzed with the Abi Prism 7000 SDS software (Applied Biosystems). The following PCR conditions were applied: 50°C for 2 min, 95°C for 10 min, and 40 amplification cycles (95°C for 15 s and 60°C for 60 s).

In order to determine the efficiency of each TaqMan gene expression assay, standard curves were generated by serial dilution of cDNA, and quantitative evaluations of target and housekeeping gene levels were obtained by measuring threshold cycle numbers (Ct). A relative quantitative analysis was performed, using the 2–ΔΔCt value, where ΔCt = Ct (target)—Ct (endogenous control) and ΔΔCt = ΔCt (sample)—ΔCt (calibrator). Beta actin was used as an endogenous control, and the sample with the lowest expression was used as a calibrator (syndecan-3 in HT-29).

### Gene Expression of Human Sulfatases by RT-PCR

PANC-1, HT-29, MDA-MB-231, and MCF-7 cells were seeded in 6-well plates (5 × 10^5^ cells per well) and cultured overnight in a CO_2_ incubator. Total RNA was extracted using an RNA isolation kit (Macherey-Nagel) according to the manufacturer's instructions. RNA was quantified by spectrophotometry at 260 and 280 nm and verified by agarose gel electrophoresis. The same quantity of RNA for every cell line was loaded on the gel. One-step RT-PCR (QIAGEN) was applied for retrotranscription and human cDNA amplification of SULF-1 (393 pb) and SULF-2 (434 pb). The following oligonucleotides were used as primers: SULF-1 primers, 5'-ACTTCCACTGCCTGCGTAATGA-3′ (sense) and 5′-ATGAACGCTTTGAGGCTAGGCA-3′ (antisense); SULF-2 primers, 5′-CCCAGAAGCTCACAAAGGAAAACG-3′ (sense) and 5′-AATGTCCACAACTGCGAGGGAT-3′ (antisense).

The following PCR conditions were applied: for SULF-1, 30 denaturing cycles at 94°C for 60 s, annealing at 58°C for 60 s, and extension at 72°C for 90 s; for SULF-2, 30 denaturing cycles at 94°C for 60 s, annealing at 54°C for 60 s, and extension at 72°C for 60 s. Glyceraldehyde-3-phosphate dehydrogenase (GAPDH) was used as experimental control. Signals were detected using Image LAS4010 (GE Healthcare). Densitometry analysis was carried out using ImageJ software. The value 100% corresponds to GAPDH gene expression for each cell line. The experiment was performed twice. *P* values were calculated using a one-tailed Student *t*-test and GraphPad Prism 5.0 software.

### Expression of Sulf-1

HT-29, PANC-1, MDA-MB-231, and MCF-7 cells were seeded in 6-well plates (1.5 × 10^6^ cells per well), previously coated with 10 μg/ml plasma fibronectin, and maintained overnight in a CO_2_ incubator. Cells were lysed according to the antibody supplier's instructions (Abcam). Total proteins (20 μl/lane) were separated with a 12% sodium dodecyl sulfate-polyacrylamide gel electrophoresis (SDS-PAGE) and transferred to a nitrocellulose membrane (GE Healthcare). The membrane was saturated with 5% w/v nonfat dry milk in PBS containing 0.1% Tween20 for 1 h at room temperature and then incubated with specific antibodies [rabbit polyclonal to sulfatase 1/SULF-1 antibody (1 μg/ml, Abcam), and mouse anti-GAPDH monoclonal antibody (1 μg/ml, Invitrogen)]. After washing, the membrane was incubated with horseradish peroxidase-conjugated anti-rabbit IgG (1:2,000, Cell Signaling) in the case of anti-sulfatase 1/SULF-1 antibody and with horseradish peroxidase-conjugated anti-mouse immunoglobulin G (IgG) (1:10,000, ThermoFisher). Signals were detected using Image LAS4010 (GE Healthcare). Densitometry analysis was carried out using ImageJ software. The value 100% corresponds to average GAPDH protein expression for the four cell lines. The experiment was performed three times. *P* values were calculated using a parametric, unpaired Student *t*-test, and GraphPad Prism 5.0 software.

### Surface Plasmon Resonance (SPR) Experiments

Experiments were performed on a Biacore T100 instrument (GE Healthcare). All materials were purchased from GE Healthcare unless otherwise specified. Full-length recombinant human HSPG were purchased from R&D Systems. Syndecan-3, syndecan-4, and glypican-3 were obtained from the mouse myeloma cell line (NS0), and glypican-4 was obtained from the Chinese Hamster Ovary cell line. The activity of syndecan-4, glypican-3, and glypican-4 was measured by the supplier as the ability of the immobilized protein to bind FGF-basic. The activity of syndecan-3 was measured by the supplier as the ability of the immobilized protein to inhibit adhesion of Saos-2 human osteosarcoma cells to human fibronectin.

Eight-mer and nine-mer oligosaccharides S00 (GlcNAc-GlcA)_4_ α-paranitrophenyl, S04 (GlcNS-GlcA)_4_ α-paranitrophenyl, S06a (GlcA-GlcNS)_2_-(GlcA-GlcNS,6S)_2_-GlcA α-paranitrophenyl (9-mer), and S06b GlcNS-GlcA-GlcNS-IdoA,2S-GlcNS-IdoA,2S-GlcNS-GlcA α-paranitrophenyl were purchased from Iduron. In all oligosaccharides, the units were linked together by α (1–4) bonds only and carry a paranitrophenyl group. S12 (ΔHexA,2S α1-4 GlcNS,6S)_3_ (9-mer) was from Amsbio, and its first glycoside is unsaturated.

NT4-biotin was captured on a CM5 sensor chip where streptavidin had previously been immobilized by standard amine coupling. Briefly, the sensor chip surface was activated with a mixture of 0.1 M 1-ethyl-3(3-dimethylaminopropyl)-carbodiimide (EDC) and 0.4 M N-hydroxyl succinimide (NHS) for 7 min at a flow rate of 5 μl/min. Streptavidin was injected over the surface for 7 min, and finally, 1 M ethanolamine pH 8.5 was used to block any activated carboxyl groups. NT4-biotin, diluted in HBS-EP+ (Hepes 10 mM, NaCl 150 mM, EDTA 3.4 mM, 0.05% p20, pH 7.4) to 30 μg/ml, was injected for 2 min at a flow rate of 10 μl/min.

HSPG and oligosaccharides were diluted to different concentrations in HBS-EP+ and then injected over immobilized NT4 peptides. The sensor chip surface was regenerated with a short pulse of 10 mM NaOH/0.5 M NaCl 5 min after the end of the injections.

Kinetics were analyzed with the Biacore T100 evaluation 1.1.1 software using the 1:1 Langmuir model to fit the curves.

### Modeling of NT4-Sulfated Oligosaccharide Complex

NT4 was modeled as extended conformation structure using PyMOL (The PyMOL Molecular Graphics System, Version 1.4, Schrödinger, LLC) and refined by energy minimization with the Gromacs package (27) and Amber force field (28). The molecule was centered in a triclinic box with at least 10-Å distance from the solute to the periodic box border; the box was filled with TIP3P water model, and the system was neutralized by adding counterions. A new force field entry was created for lysine in the scaffold by reparameterization of the standard lysine residue from the Amber library, taking covalent bonding of the side-chain amine into account. The peptide was linked to available amines of the scaffold. The three-dimensional (3D) structure of the 8-mer heparin oligosaccharide was derived from the canonical helical structure of heparin (PDB ID 1HPN, ^1^C_4_ conformer) (29). The GLYCAM06 force field parameters (30) were used for GAGs.

## Results

In previous papers, we reported NT4 binding and internalization into different cancer cell lines by immunofluorescence and flow cytometry (11, 13, 14, 19). In previous confocal microscopy experiments, NT4 conjugated with biotin (NT4-biotin) already proved to be completely internalized only after 2 h at 37°C (14, 16). Degradation of NT4-biotin by living cells was previously assessed by mass spectrometry and showed that the molecule was still stable after 24 h (14). NT4 binding and internalization into those cancer cells or tissues were completely inhibited by heparin and HS (13, 19). We also demonstrated that NT4 binds to heparin and HS with high affinity and to CS with lower affinity (13).

**Figure 1:**
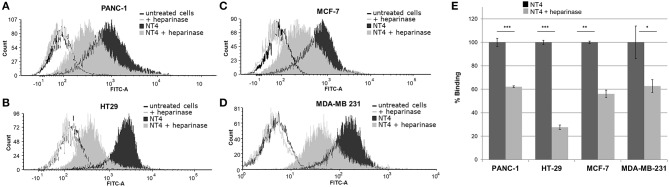
**(A–D)** NT4 binding to PANC-1, HT-29, MCF-7, and MDA-MB-231 cancer cells before and after heparinase I/III treatment, tested by flow cytometry. **(E)** Variation of binding of NT4 after heparinase treatment, 100% (dark histogram) is the baseline binding of NT4 to the different cell lines ***p < 0.001, **p < 0.01, *p < 0.05 by Student's *t*-test.

To further assess the specificity of binding of the NT4 peptide to HSPG in HT-29 colon adenocarcinoma, PANC-1 pancreas adenocarcinoma, and MDA-MB-231 and MCF-7 breast cancer human cell lines, we first treated the cells with the heparinase I/III blend that removes HS from proteoglycans. We then incubated the cells with NT4. Flow cytometry analysis showed that NT4 binding to cancer cells treated with heparinase was much lower than to control cells (Figure 1).

### Gene Expression of Glypicans and Syndecans in HT-29, PANC-1, MDA-MB-231, and MCF-7 Cancer Cells

Glypican and syndecan levels have recently been studied with a view to defining new tumor markers or prognostic tools (6, 31). Elevated levels of glypican-1 are found in pancreas carcinoma where increased expression is associated with poor prognosis (32). Levels of glypican-1 and syndecan-2 are also increased in colorectal cancer (1). Breast cancer was found to upregulate glypican-1 (33–35) and syndecan-4 (36) and to downregulate glypican-3 (37), while loss of glypican-3 promotes tumor proliferation and metastasis (38). Glypican-2 is upregulated in neuroblastoma and associated with poor overall survival (1). The roles of glypican-4 and syndecan-3 in tumors are still underexplored.

**Figure 2:**
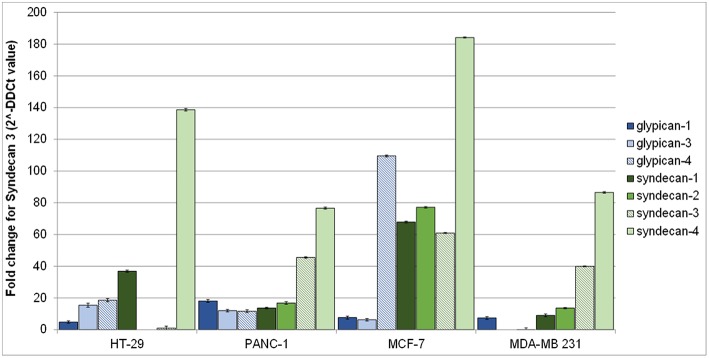
Gene expression of human glypicans (shades of blue) and syndecans (shades of green) in HT-29, PANC-1, MCF-7, and MDA-MB-231 human cancer cell lines determined by qRT-PCR and normalized against β-actin. Results are reported as fold change for syndecan-3 in HT-29.

Figure 2 shows syndecans and glypicans expression in HT-29, PANC-1, MDA-MB-231, and MCF-7 as analyzed by qRT-PCR.

Expression of syndecans (Figure 2, shades of green) was generally higher than that of glypicans (Figure 2, shades of blue). Among syndecans, syndecan-4 was the most expressed in all cell lines, followed by syndecan-3 in MCF-7, MDA-MB-231, and PANC-1 cells. Among glypicans, glypican-4 was the most expressed, but only in MCF-7 cells (Figure 2).

### Sulfatases Modulate NT4 Binding on Cancer Cells

Human sulfatase 1 (hSULF-1) and human sulfatase 2 (hSULF-2) are extracellular enzymes that remove 6-O-sulfate groups from HS chains. Modified expression of both sulfatases, particularly SULF-1, has been associated with different cancers (38). By hydrolyzing 6-O-sulfate groups, hSULF-1 and hSULF-2 modulate binding of HS-binding proteins, such as growth factors and cytokines, and, finally, have effects on cell signaling (38). For example, hSULF-1, acting on HS, reduces the formation of the FGF2–FGFR–HS complex and consequently impairs FGF2 signaling (39).

**Figure 3:**
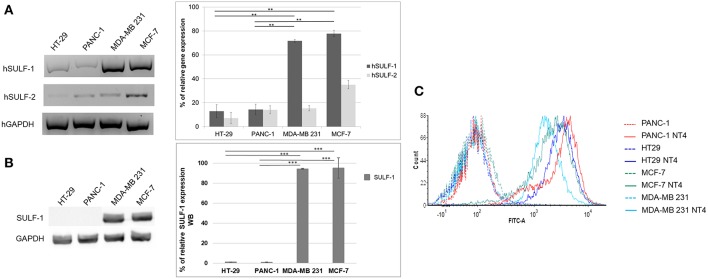
**(A)** Gene expression of hSULF-1 and hSULF-2 in HT-29, PANC-1, MCF-7, and MDA-MB-231 cell lines analyzed by RT-PCR. The GAPDH gene was tested as endogenous control. Histograms represent percentage of gene expression compared to GAPDH (GAPDH is 100%). **(B)** SULF-1 expression analyzed by Western blot in the same cell lines. Histograms represent percentage of SULF-1 expression compared to GAPDH (GAPDH is 100%). Significance of the differences was calculated using Student's *t*-test and GraphPad, **p < 0.01; ***p < 0.001. **(C)** NT4 binding analyzed by flow cytometry in HT-29, PANC-1, MCF-7, and MDA-MB-231.

Figure 3A shows the relative abundance of mRNA of hSULF-1 and hSULF-2 in HT-29, PANC-1, MCF-7, and MDA-MB-231 cells as measured by RT-PCR. The two sulfatases were expressed very differently in the different cell lines. SULF-1 protein expression was also measured in the same cell lines using a specific anti-SULF-1 antibody (Figure 3B). PANC-1 and HT-29 cells showed much lower expression of sulfatases, which implies that their sulfated GAG chains retain more 6-O-sulfate groups than cancer cells with higher expression of sulfatases, such as MCF-7 and MDA-MB-231.

The pattern of NT4 cell binding detected by flow cytometry (Figure 3C) suggests that cells expressing lower levels of sulfatases, particularly SULF-1, such as PANC-1 and HT-29, bind NT4 better than the others. The higher presence of the 6-O-sulfate groups is therefore correlated with higher binding of NT4 to those cell lines.

### Affinity of NT4 for Recombinant HSPG and Sulfated GAGs

**Figure 4:**
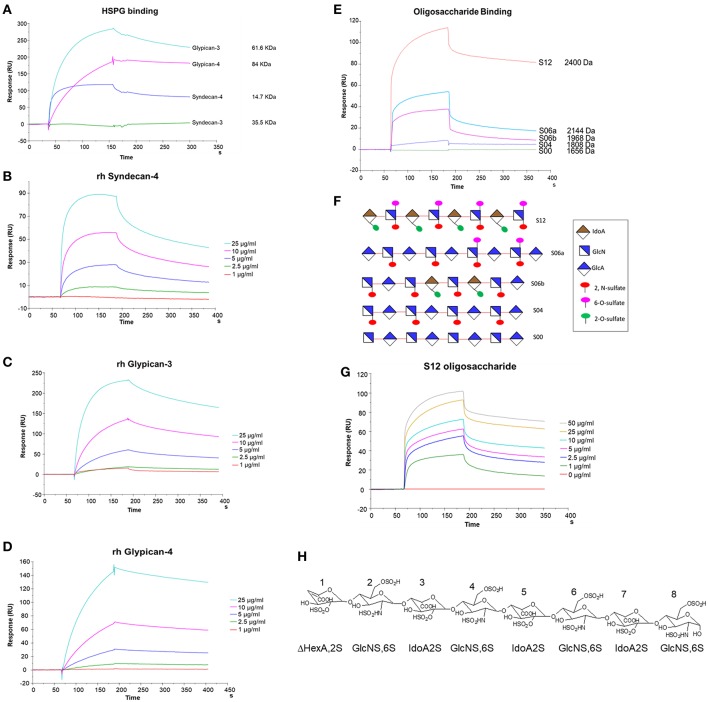
SPR analysis of rHSPG and oligosaccharide binding to NT4. **(A)** rHSPG binding (25 μg/mL) to immobilized NT4. **(B–D)** Affinity of rHSPG for NT4. **(E)** Oligosaccharide (100 μg/ml) binding to surface immobilized NT4. **(F)** Schematic representation of oligosaccharides with sulfation sites. **(G)** Affinity of S12 sulfated oligosaccharide binding to NT4. **(H)** Structure of S12.

We used SPR to measure the affinity of NT4 binding to recombinant syndecans and glypicans, selected among those highly expressed by HT-29, PANC-1, MDA-MB-231, and MCF-7 cancer cell lines. We found that NT4 does not bind syndecan-3, whereas it binds syndecan-4, glypican-3, and glypican-4 (Figures 4A–D) with different affinities, the affinity of both glypicans being five times greater than that of syndecan-4. SPR analysis also enabled kinetic evaluation of NT4 binding to HSPG, showing different kinetic rates of association and dissociation (Table 1).

**Table 1:** Kon, koff, and KD of recombinant glypicans and syndecans and oligosaccharides.

	**Ka (M^**−1**^s^**−1**^)**	**Kd (s^**−1**^)**	**KD (M)**
Syndecan-4	0.901E+4	23.16E−4	2.570E−7
Glypican-3	2.392E+4	14.33E−4	5.989E−8
Glypican-4	0.871E+4	6.719E−4	7.708E−8
S06a	0.106E+4	25.75E−4	2.427E−6
S06b	0.149E+4	40.39E−4	2.700E−6
S12	0.353E+4	9.126E−4	2.578E−7

Binding of NT4 to synthetic oligosaccharides carrying different sulfation patterns was also analyzed. We used 8-mer and 9-mer oligosaccharides with different sulfation patterns: no sulfation in oligosaccharide S00, 4 N-sulfate groups in S04, 6 sulfate groups in S06a including 4 N-sulfates and 2 6-sulfates, 6 sulfate groups in S06b including 4 N-sulfates and 2 2-O-sulfates, and, finally, 12 sulfate groups in S12, 4 in 6-O-position, 4 in 2-O, and 4 in N. We observed that the more sulfate groups there were, the higher was the affinity of the oligosaccharide for the peptide. We also found a correlation between sulfation in position 6 of oligosaccharides and NT4 binding affinity. Indeed, S06a, which carries the same number of sulfates as S06b, bound NT4 better by virtue of having two 6-O-sulfates (Figure 4). The best-binding oligosaccharide was S12, which carries repeated 6-O-sulfates, like S06a, but the 6-O-sulfates in S12 are alternated with 2-O or N-sulfates, making binding more stable (Table 1).

### Graphical Model of Interaction of NT4 and a Sulfated Oligosaccharide

NT4 was modeled with PyMol and refined by energy minimization. The 3D structure of the positively charged stretch of the NT4 peptide sequence (K6PRRP10), previously demonstrated to be critical for heparin binding (19), resulted in an extended conformation that lowers steric hindrance between rigid prolines and their preceding amino acids bearing a large side chain. This conformation gives rise to a triangular pattern formed by the charged termini of K6, R8, and R9, with 6–8 and 8–9 distances of ~12 Å and an angle of ~130° between residues 6–8–9.

The 8-mer oligosaccharide was chosen for the *in silico* study on the basis of the experimental result obtained with flow cytometry that identified S12 (12 sulfate groups in an 8-mer) as the best-binding oligosaccharide, and its 3D structure was derived from the canonical helical structure of heparin (PDB ID 1HPN, ^1^C_4_ conformer) (29).

Previous studies showed that the binding of heparin and HS to polypeptides is ionic in nature (40–42). The charge-based interactions between the acidic substituents on the polysaccharide and basic residues on the polypeptide are reported to dominate the interface, and charges have to be in an appropriate 3D pattern (43). For example, FGF1 proved to prefer a specific pattern of sulfate groups in a specific spatial distribution (44). Following such evidences, a matching between charge clusters was attempted by mean of 3D molecular graphics.

**Figure 5:**
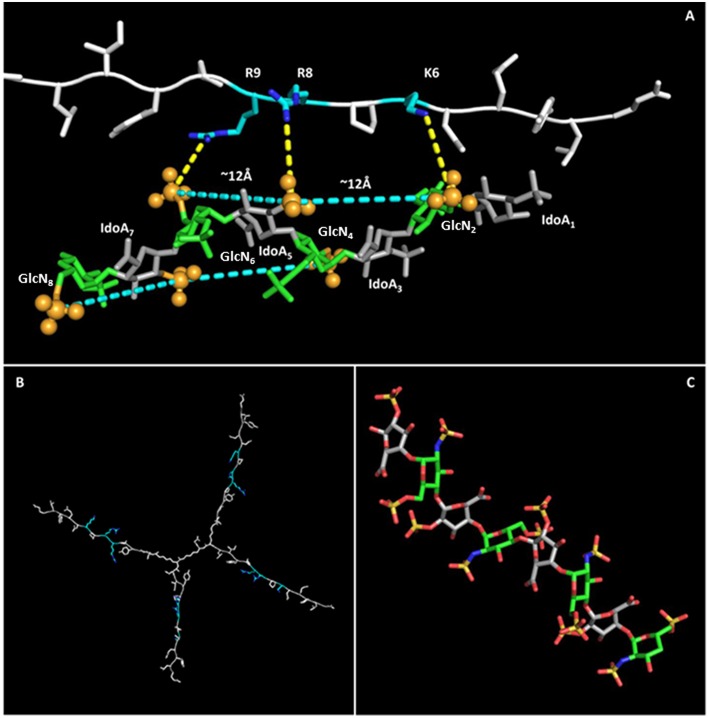
NT4-sulfated oligosaccharide hypothetical complex. **(A)** Model of NT4 complexed with the 8-mer sulfated oligosaccharide. Clusters of sulfates on both sides of the helical structure of the oligosaccharide are identified by pale blue lines, with the sulfates involved represented as spheres. Polar interactions between the positive charges on peptide residues and sulfate negative clusters are drawn as dashed yellow lines. **(B)** Model of NT4 structure with KPRR motifs in pale blue. **(C)** Structure of the sulfated oligosaccharide (29).

Indeed, the sulfates of GlcNS_i−3_-IdoA2S_i_-GlcNS6S_i+1_ (corresponding to GlcN_2_-IdoA_5_-GlcN_6_ and GlcN_4_-IdoA_7_-GlcN_8_), lying on the same side of the helix, form a pattern with distances and angles coherent with those of charged side chains of KPRR, and a specific geometry of interaction of charges is suggested (yellow dashed lines in Figure 5). Similar results hold for the ^1^C_4_ and ^2^S_0_ cyclic forms of the oligosaccharide. On an 8-mer saccharide, this pattern is found twice on opposite sides of the helix, possibly interacting with two different NT4 peptide arms.

This interaction model also explains the almost total loss of binding for S04 (N-sulfates only), where alternate side sulfates are unable to form any negative charge cluster (Figure 5) that could fit with the positive cluster of NT4.

The *in silico* modeling provides a theoretical picture of the interaction that can help in understanding the binding activity of NT4. In particular, the fact that the oligosaccharide has two negative clusters on opposite sides of the molecule could reinforce the hypothesis of multiple binding with NT4.

## Discussion

HSPG are synthesized by most animal cells, but due to the variable composition and sulfation of their GAG chains, their ability to interact with specific ligands may be modulated under different physiological and pathological conditions, including cancer. Tumor stroma is composed of the ECM, including proteoglycans, fibronectin, collagen, cytokines, and growth factors. Cells that populate the tumor stroma, like immune system cells, fibroblasts, and endothelial cells, together with tumor cells, can modify the stroma as the tumor evolves. The ECM of the tumor stroma is very different from that of normal tissues (1) due to tumor remodeling that also triggers tumor invasiveness (1). HSPG accumulate in remodeled stroma and are, in turn, modified on their glycosidic chains by tumor-dependent glycosyltransferases, sulfotransferases, sulfatases, and heparanases (6, 45). The presence and amount of these GAG-related enzymes help identify high-risk tumors and develop targeting therapies (46). In colon tumors, for example, significant upregulation of extracellular sulfatases SULF-1/2 has been observed and may indicate general alteration of HS 6-O-sulfation patterns in colon tumors (47).

As discussed in the introduction, hundreds of different extracellular regulatory proteins, such as growth factors, chemokines, and morphogens, also involved in cancer, interact with the GAG portion of HSPG, requiring specific glycosides sequences and sulfation patterns (23).

The peculiar post-translationally regulated variability of HSPG has made it difficult to study their activity in cancer cell biology.

NT4 is already known to have major effects on cancer cells, such as inhibition of migration and invasion of ECM induced by FGF (20).

We examined the expression of syndecans and glypicans in a panel of cancer cell lines that NT4 binds. The binding affinity of NT4 with human rHSPG expressed by these cells was then analyzed by SPR. NT4 did not bind syndecan-3, but it bound glypican-3 and -4, and also syndecan-4, but with one fifth of the affinity shown for glypicans.

Glypicans and syndecans have different GAG chains: glypicans only carry HS chains, whereas syndecans-2 and 4 have HS chains and syndecans-1 and 3 have HS and CS chains (4, 48). Besides, HS posttranslational modifications occur in clusters, i.e., HS has some domains that are more densely sulfated than others. For example, the FGF binding domain that has 2-, 6-, and N-sulfation, carries seven sulfated groups in five residues, whereas the anti-thrombin binding domain contains six sulfated groups in five residues. In contrast, CS has more homogeneously sulfated patterns with long tracts carrying an average of four sulfates every five residues (49).

The NT4 affinity profile is therefore consistent with our previous results that showed a preference of the peptide for HS chains featuring patches of dense sulfation, compared to CS (49).

Another important finding regarding NT4 recognition of sulfated GAG chains came from the analysis of sulfatase expression in the same panel of human cancer cell lines. NT4 binding to the cancer cell membrane was inversely correlated with expression of sulfatases. NT4 binding was higher in cell lines with lower expression of sulfatases, particularly SULF-1, i.e., HT-29 and PANC-1, confirming the determinant role of 6-O-sulfate groups for recognition by NT4.

Using 8-mer and 9-mer HS oligosaccharides with analog disaccharide composition and different sulfation sites, a possible recognition motif was identified that includes repeated 6-O-sulfates alternating with N- and/or 2-O-sulfates. This finding is again consistent with the preference of NT4 for HS more than for CS. CS carries GAG chains with 2-O-sulfates and 4-O-sulfates, whereas HS has 6-O-sulfates alternating with N- or 2-O-sulfates.

The possible structure of the NT4-sulfated oligosaccharide complex was then reconstructed by molecular modeling, taking into account our information on amino acids in NT4 sequences, i.e., KPRR, previously demonstrated to be essential for heparin and HS binding (13, 19). The modeling showed that the distance between the crucial positive residues of NT4 is completely compatible with ionic interaction with sulfates on the oligosaccharide. Moreover, assuming a helical structure of the oligosaccharide, which is considered usual for sulfated oligosaccharides, sulfate groups lying on opposite sides of the helix can interact with positive residues on two peptide sequences of the branched structure, thus favoring multivalent binding, and explaining the high affinity and selectivity of NT4 for highly sulfated GAGs. Being a branched peptide, NT4 can give multiple binding to repeated domains on the same GAG chain or on different GAG chains of the same HSPG, improving binding affinity. Specificity of GAG ligand binding, which allows formation of the GAG–ligand–receptor complex that triggers signal transduction, is mediated by multivalent electrostatic interactions between GAGs and growth factors or proteins of the ECM. The presence of binding sites of growth factors and proteins on GAG chains is no longer disputed, and the exact structure and motifs of the recognition patterns are being explored (23, 50, 51).

NT4 and possibly newly selected branched peptides can be designed and used to unravel the exact structure of binding sites on GAG chains. These tools will be essential probes for reconstructing binding sites for cancer-involved ligands on GAGs, paving the way for new cancer detection and treatment options.

## Data Availability

All datasets generated for this study are included in the manuscript and/or the supplementary files.

## Author Contributions

CF, JB, LD, APi, APa, and LB conceived and designed the experiments. AB designed and performed the modeling experiments. EK performed the qRT PCR experiments. JB, GR, EM, and LD performed flow cytometry, SPR, and Western blot. CF and LB wrote the paper. CF supervised the project. All authors read and approved the final manuscript.

### Conflict of Interest Statement

The authors declare that the research was conducted in the absence of any commercial or financial relationships that could be construed as a potential conflict of interest.

## References

[1] Theocharis AD Karamanos NK. Proteoglycans remodeling in cancer: underlying molecular mechanisms. Matrix Biol. (2017) 75–6:220–59. 10.1016/j.matbio.2017.10.008 29128506

[2] Karamanos NK Piperigkou Z Theocharis AD Watanabe H Franchi M Baud S. Proteoglycan chemical diversity drives multifunctional cell regulation and therapeutics. Chem Rev. (2018) 118:9152–232. 10.1021/acs.chemrev.8b00354 30204432

[3] Bernfield M Gotte M Park PW Reizes O Fitzgerald ML Lincecum J. Functions of cell surface heparan sulfate proteoglycans. Annu Rev Biochem. (1998) 68:729–77. 10.1146/annurev.biochem.68.1.729 10872465

[4] Iozzo RV Schaefer L. Proteoglycan form and function: a comprehensive nomenclature of proteoglycans. Matrix Biol. (2015) 42:11–55. 10.1016/j.matbio.2015.02.003 25701227 PMC4859157

[5] Couchman JR. Transmembrane signaling proteoglycans. Annu Rev Cell Dev Biol. (2010) 26:89–114. 10.1146/annurev-cellbio-100109-104126 20565253

[6] Lanzi C Zaffaroni N Cassinelli G. Targeting heparan sulfate proteoglycans and their modifying enzymes to enhance anticancer chemotherapy efficacy and overcome drug resistance. Curr Med Chem. (2017) 24:2860–86. 10.2174/0929867324666170216114248 28215163

[7] Beauvais DM Jung O Yang Y Sanderson RD Rapraeger AC Syndecan-1. (Cd138) suppresses apoptosis in multiple myeloma by activating igf1 receptor: prevention by synstatin igf1r inhibits tumor growth. Cancer Res. (2016) 76:4981–93. 10.1158/0008-5472.CAN-16-0232 27364558 PMC5010496

[8] Rapraeger AC. Synstatin: a selective inhibitor of the syndecan-1-coupled igf1r-αvβ3 integrin complex in tumorigenesis and angiogenesis. FEBS J. (2013) 280:2207–15. 10.1111/febs.12160 23375101 PMC3651771

[9] Bracci L Falciani C Lelli B Lozzi L Runci Y Pini A. Synthetic peptides in the form of dendrimers become resistant to protease activity. J Biol Chem. (2003) 278:46590–5. 10.1074/jbc.M308615200 12972419

[10] Falciani C Lozzi L Pini A Corti F Fabbrini M Bernini A. Molecular basis of branched peptides resistance to enzyme proteolysis. Chem Biol Drug Des. (2007) 69:216–21. 10.1111/j.1747-0285.2007.00487.x 17441908

[11] Falciani C Fabbrini M Pini A Lozzi L Lelli B Pileri S. Synthesis and biological activity of stable branched neurotensin peptides for tumor targeting. Mol Cancer Ther. (2007) 6:2441–8. 10.1158/1535-7163.MCT-07-0164 17766836

[12] Falciani C Brunetti J Pagliuca C Menichetti S Vitellozzi L Lelli B. Design and *in vitro* evaluation of branched peptide conjugates: turning nonspecific cytotoxic drugs into tumor-selective agents. Chem Med Chem. (2010) 5:567–74. 10.1002/cmdc.200900527 20222099

[13] Brunetti J Depau L Falciani C Gentile M Mandarini E Riolo G. Insights into the role of sulfated glycans in cancer cell adhesion and migration through use of branched peptide probe. Sci Rep. (2016) 6:27174. 10.1038/srep27174 27255651 PMC4891694

[14] Falciani C Lelli B Brunetti J Pileri S Cappelli A Pini A. Modular branched neurotensin peptides for tumor target tracing and receptor-mediated therapy: a proof-of-concept. Curr Cancer Drug Targets. (2010) 10:695–704. 10.2174/156800910793605875 20578987

[15] Falciani C Accardo A Brunetti J Tesauro D Lelli B Pini A. Target-selective drug delivery through liposomes labeled with oligobranched neurotensin peptides. Chem Med Chem. (2011) 6:678–85. 10.1002/cmdc.201000463 21370475

[16] Brunetti J Falciani C Lelli B Minervini A Ravenni N Depau L. Neurotensin branched peptide as a tumor-targeting agent for human bladder cancer. Biomed Res Int. (2015) 2015:173507. 10.1155/2015/173507 25984525 PMC4423026

[17] Brunetti J Pillozzi S Falciani C Depau L Tenori E Scali S. Tumor-selective peptide-carrier delivery of paclitaxel increases *in vivo* activity of the drug. Sci Rep. (2015) 5:17736. 10.1038/srep17736 26626158 PMC4667195

[18] Brunetti J Riolo G Gentile M Bernini A Paccagnini E Falciani C. Near-infrared quantum dots labelled with a tumor selective tetrabranched peptide for *in vivo* imaging. J Nanobiotechnol. (2018) 16:21. 10.1186/s12951-018-0346-1 29501065 PMC5834876

[19] Falciani C Brunetti J Lelli B Ravenni N Lozzi L Depau L. Cancer selectivity of tetrabranched neurotensin peptides is generated by simultaneous binding to sulfated glycosaminoglycans and protein receptors. J Med Chem. (2013) 56:5009–18. 10.1021/jm400329p 23713525

[20] Bracci L Mandarini E Brunetti J Depau L Pini A Terzuoli L. The GAG-specific branched peptide NT4 reduces angiogenesis and invasiveness of tumor cells. PLoS ONE. (2018) 13:e0194744. 10.1371/journal.pone.0194744 29566097 PMC5864057

[21] Xu R Ori A Rudd TR Uniewicz KA Ahmed YA Guimond SE. Diversification of the structural determinants of fibroblast growth factor–heparin interactions: implications for binding specificity. J Biol Chem. (2012) 287:40061–73. 10.1074/jbc.M112.398826 23019343 PMC3501079

[22] Pye DA Vives RR Turnbull JE Hyde P Gallagher JT. Heparan sulfate oligosaccharides require 6-O-sulfation for promotion of basic fibroblast growth factor mitogenic activity. J Biol Chem. (1998) 273:22936–42. 10.1074/jbc.273.36.22936 9722514

[23] El Masri R Seffouh A Lortat-Jacob H Vivès RR. The “in and out” of glucosamine 6-O-sulfation: the 6th sense of heparan sulfate. Glycoconj J. (2017) 34:285–98. 10.1007/s10719-016-9736-5 27812771

[24] Seffouh A Milz F Przybylski C Laguri C Oosterhof A Bourcier S. HSulf sulfatases catalyze processive and oriented 6-O-desulfation of heparan sulfate that differentially regulates fibroblast growth factor activity. Faseb J. (2013) 27:2431–9. 10.1096/fj.12-226373 23457216

[25] Lu J Auduong L White ES Yue X. Up-regulation of heparan sulfate 6-O-sulfation in idiopathic pulmonary fibrosis. Am J Respir Cell Mol Biol. (2014) 50:106–14. 10.1165/rcmb.2013-0204OC 23962103 PMC3930936

[26] Ai X Do AT Lozynska O Kusche-Gullberg M Lindahl U Emerson CP. QSulf1 remodels the 6-O sulfation states of cell surface heparan sulfate proteoglycans to promote Wnt signaling. J Cell Biol. (2003) 162:341–51. 10.1083/jcb.200212083 12860968 PMC2172803

[27] Berendsen HJC Spoel DVD Drunen RV Gromacs: a message-passing parallel molecular dynamics implementation. Comp Phys Comm. (2012) 91:43–56. 10.1016/0010-4655(95)00042-E

[28] Sorin EJ Pande VS. Exploring the helix-coil transition *via* all-atom equilibrium ensemble simulations. Biophys J. (2005) 88:2472–93. 10.1529/biophysj.104.051938 15665128 PMC1305346

[29] Mulloy B Forster MJ Jones C Davies DB NMR and molecular-modelling studies of the solution conformation of heparin. Biochem J. (1993) 293:849–58. 10.1042/bj2930849 8352752 PMC1134446

[30] Kirschner KN Yongye AB Tschampel SM González-Outeiriño J Daniels CR Foley BL. GLYCAM06: a generalizable biomolecular force field. Carbohydr J Comput Chem. (2008) 29:622–55. 10.1002/jcc.20820 17849372 PMC4423547

[31] Wu Q Pi L Le Trinh T Zuo C Xia M Jiao Y. A novel vaccine targeting glypican-3 as a treatment for hepatocellular carcinoma. Mol Ther. (2017) 25:2299–308. 10.1016/j.ymthe.2017.08.005 28865999 PMC5628867

[32] Melo SA Luecke LB Kahlert C Fernandez AF Gammon ST Kaye J. Glypican-1 identifies cancer exosomes and detects early pancreatic cancer. Nature. (2015) 523:177–82. 10.1038/nature14581 26106858 PMC4825698

[33] Huang G Ge G Izzi V Greenspan DS. α3 chains of type V collagen regulate breast tumour growth *via* glypican-1. Nat Commun. (2017) 8:14351. 10.1038/ncomms14351 28102194 PMC5253704

[34] Aikawa T Whipple CA Lopez ME Gunn J Young A Lander AD. Glypican-1 modulates the angiogenic and metastatic potential of human and mouse cancer cells. J Clin Invest. (2008) 118:89–99. 10.1172/JCI32412 18064304 PMC2117766

[35] Matsuda K Maruyama H Guo F Kleeff J Itakura J Matsumoto Y Glypican-1 is over- expressed in human breast cancer and modulates the mitogenic effects of multiple heparin-binding growth factors in breast cancer cells. Cancer Res. (2001) 61:5562–9. 11454708

[36] Baba F Swartz K van Buren R Eickhoff J Zhang Y Wolberg W. Syndecan-1 and syndecan-4 are overexpressed in an estrogen receptor-negative, highly proliferative breast carcinoma subtype. Breast Cancer Res Treat. (2006) 98:91–8. 10.1007/s10549-005-9135-2 16636895

[37] Xiang YY Ladeda V Filmus J. Glypican-3 expression is silenced in human breast cancer. Oncogene. (2001) 20:7408–12. 10.1038/sj.onc.1204925 11704870

[38] Han S Ma X Zhao Y Zhao H Batista A Zhou S. Identification of glypican-3 as a potential metastasis suppressor gene in gastric cancer. Oncotarget. (2016) 7:44406–16. 10.18632/oncotarget.9763 27259271 PMC5190106

[39] Hammond E Khurana A Shridhar V Dredge K. The role of heparanase and sulfatases in the modification of heparan sulfate proteoglycans within the tumor microenvironment and opportunities for novel cancer therapeutics. Front Oncol. (2014) 4:195. 10.3389/fonc.2014.00195 25105093 PMC4109498

[40] Fromm JR Hileman RE Caldwell EE Weiler JM Linhardt RJ. Pattern and spacing of basic amino acids in heparin binding sites. Arch Biochem Biophys. (1997) 343:92–100. 10.1006/abbi.1997.0147 9210650

[41] Sarkar A Desai UR. A simple method for discovering druggable, specific glycosaminoglycan–protein systems. Elucidation of key principles from heparin/heparan sulfate-binding proteins. PLoS ONE. (2015) 10:e0141127. 10.1371/journal.pone.0141127 26488293 PMC4619353

[42] Lindahl U Kjellén L. Pathophysiology of heparan sulphate: many diseases, few drugs. J Intern Med. (2013) 273:555–71. 10.1111/joim.12061 23432337

[43] Mulloy B Forster MJ Application of drug discovery software to the identification of heparin-binding sites on protein surfaces: a computational survey of the 4-helix cytokines. Mol Simul. (2008) 34:481–9. 10.1080/08927020701784754

[44] Mulloy B. The specificity of interactions between proteins and sulfated polysaccharides. Anais Acad Bras Cienc. (2005) 77:651. 10.1590/S0001-37652005000400007 16341442

[45] Lim HC Multhaupt HA Couchman JR. Cell surface heparan sulfate proteoglycans control adhesion and invasion of breast carcinoma cells. Mol Cancer. (2015) 14:15. 10.1186/s12943-014-0279-8 25623282 PMC4326193

[46] Subbarayan K Seliger B. Tumor-dependent effects of proteoglycans and various glycosaminoglycan synthesizing enzymes and sulfotransferases on patients' outcome. Curr Cancer Drug Targets. (2019) 19:210–21. 10.2174/1568009618666180706165845 29984655

[47] Suhovskih AV Aidagulova SV Kashuba VI Grigorieva EV. Proteoglycans as potential microenvironmental biomarkers for colon cancer. Cell Tissue Res. (2015) 361:833–44. 10.1007/s00441-015-2141-8 25715761

[48] Sarrazin S Lamanna WC Esko JD. Heparan sulfate proteoglycans. Cold Spring Harb Perspect Biol. (2011) 3:a004952. 10.1101/cshperspect.a004952 21690215 PMC3119907

[49] Varki A Cummings RD Esko JD Freeze HH Stanley P Bertozzi CR editors. Essentials of Glycobiology, 2nd Edn. Cold Spring Harbor, NY: Cold Spring Harbor Laboratory Press (2009). p. Chapter 26. 20301239

[50] Connell BJ Sadir R Baleux F Laguri C JP Kleman Luo L. Heparan sulfate differentially controls CXCL12α- and CXCL12γ-mediated cell migration through differential presentation to their receptor CXCR4. Sci Signal. (2016) 9:ra107. 10.1126/scisignal.aaf1839 27803285

[51] Köhling S Blaszkiewicz J Ruiz-Gómez G Fernández-Bachiller MI Lemmnitzer K Panitz N. Syntheses of defined sulfated oligohyaluronans reveal structural effects, diversity and thermodynamics of GAG–protein binding. Chem Sci. (2018) 10:866–78. 10.1039/C8SC03649G 30774881 PMC6346292

